# Modifications of Parylene by Microstructures and Selenium Nanoparticles: Evaluation of Bacterial and Mesenchymal Stem Cell Viability

**DOI:** 10.3389/fbioe.2021.782799

**Published:** 2021-12-03

**Authors:** Jana Pekarkova, Imrich Gablech, Tatiana Fialova, Ondrej Bilek, Zdenka Fohlerova

**Affiliations:** ^1^ Central European Institute of Technology, Brno University of Technology, Brno, Czechia; ^2^ Department of Microelectronics, Faculty of Electrical Engineering and Communication, Brno, University of Technology, Brno, Czechia; ^3^ Department of Chemistry and Biochemistry, Mendel University in Brno, Brno, Czechia; ^4^ Institute of Environmental Technology, VŠB—Technical University of Ostrava, Ostrava, Czechia; ^5^ Department of Biochemistry, Faculty of Medicine, Masaryk University, Brno, Czechia

**Keywords:** parylene-C, micropillars, selenium nanoparticles, biocompatibility, antimicrobial

## Abstract

Parylene-based implants or coatings introduce surfaces suffering from bacteria colonization. Here, we synthesized polyvinylpyrrolidone-stabilized selenium nanoparticles (SeNPs) as the antibacterial agent, and various approaches are studied for their reproducible adsorption, and thus the modification of parylene-C–coated glass substrate. The nanoparticle deposition process is optimized in the nanoparticle concentration to obtain evenly distributed NPs on the flat parylene-C surface. Moreover, the array of parylene-C micropillars is fabricated by the plasma etching of parylene-C on a silicon wafer, and the surface is modified with SeNPs. All designed surfaces are tested against two bacterial pathogens, *Escherichia coli* (Gram-negative) and *Staphylococcus aureus* (Gram-positive). The results show no antibacterial effect toward *S. aureus*, while some bacteriostatic effect is observed for *E. coli* on the flat and microstructured parylene. However, SeNPs did not enhance the antibacterial effect against both bacteria. Additionally, all designed surfaces show cytotoxic effects toward mesenchymal stem cells at high SeNP deposition. These results provide valuable information about the potential antibacterial treatment of widely used parylene-C in biomedicine.

## Introduction

Parylene is a commonly used polymeric protectiveness of a wide range of devices in the form of thin-film coating. Due to its excellent anticorrosion properties, it is often used as an insulator for electronic components (Marszalek et al., 2017). Its long-term stability and biocompatibility in contact with biofluids and tissues make this polymer excellent for biomedical applications (Golda-Cepa et al., 2020). To date, parylene has been used to coat implantable sensors and transducers (Zeniieh et al., 2014), and orthopedic or dental implants, such as catheters and stents (Staufert et al., 2018). Due to its natural hydrophobic character, the surface has to be chemically treated (e.g., oxygen plasma or silanization) or modified by proteins to support, for example, cell growth (Chang, 2007; Song et al., 2009). Moreover, bacterial colonization and subsequent infections remain one of the post-complications of the many hard and polymeric implants so far, and parylene is no exception (Filipović et al., 2020). Nevertheless, conventional systematic antibiotic therapy of the infected patients has been found to have low effectiveness in some cases, and adverse side effects, and increases the number of drug-resistant bacteria. Drug resistance enforces a high dose of antibiotic (ATB) application, often generating intolerable toxicity to the mammalian cells, developing new antibiotics, and searching for the advanced antimicrobial protections and coatings (Huh et al., 2011).

Currently, the utilization of non-drug antimicrobial materials and coatings has been widely studied. This introduces nanomaterials, including silver (Arya et al., 2019), gold (Piktel et al., 2021), copper (Usman et al., 2013), selenium (Huang et al., 2019), titanium oxide (Youssef et al., 2020), and zinc oxide (Janaki et al., 2015) nanoparticles; carbon nanomaterials (Azizi-Lalabadi et al., 2020); antimicrobial peptides (Raheem and Straus, 2019); or chitosan (Qi et al., 2004) which shows more or less antimicrobial effectiveness by themselves toward a specific type of bacteria. Therefore, the different strategies and combinations of various materials, geometries, and chemistry are emerging today to find enhanced antibacterial efficiency. For instance, the modification with antimicrobial agents (nanoparticles, peptides, *etc*.), oxidation, or surface functionalization has been widely studied against Gram-positive and Gram-negative bacteria (Sharma and Conrad, 2014; Bilek et al., 2020). Besides, the mechanical or physicochemical micro-/nanostructuring of the surface has been proven to enhance the antibacterial properties of some biomaterials such as titanium, tantalum, hafnium, and zirconium and its oxides (Bilek et al., 2019; Fohlerova et al., 2021; 2019). Since the polymeric materials used in medicine such as polyvinylchloride (PVC), polyurethanes (PU), silicone, and parylene also suffer from bacterial colonization and infection, the antimicrobial modification of these polymers and their biocompatibility *in vitro* and *in vivo* have been studied (Lee et al., 2013; Kupka et al., 2016). For example, three deposition modes of ZnO nanoparticle decoration of parylene-coated glass have been tested for antibacterial actions (Applerot et al., 2010). The ZnO–parylene–glass composites differed in their particle sizes, coating thickness, and depth of penetration, and demonstrated significant antibacterial activity against *E. coli* and *S. aureus*. The antimicrobial coating has been developed to inhibit bacterial growth on PVC, PU, and silicone by a coating of polymeric substrates with SeNPs *in situ* (Tran and Webster, 2013). The cytotoxicity of porous silicon materials with immobilized Au, Ag, and Cu nanoparticles has been studied on L929 fibroblasts with the statistical difference of the CuNP substrate (Solano-Umaña and Vega-Baudrit, 2017). Tetracycline nanoparticles embedded into parylene film to prevent bacteria-associated infections have been studied, and self-sterilizing the properties of the surface has been suggested (Grinberg et al., 2015). Several studies showed the biocompatibility of deposited or patterned parylene-C to the mammalian cells (Gołda et al., 2013; Delivopoulos et al., 2015; Tu et al., 2017) with suggested good performance after plasma and protein modification. To our knowledge, the micro-/nanostructured parylene-C has not been studied as the surface with potential antibacterial activity. Also, the synergetic effect of such surface with antibacterial SeNPs has not been evaluated and compared with the flat parylene-C film so far.

In this work, we present the basic experimental study of the synergistic effect of SeNPs and microstructured parylene-C surface in the form of micropillars on the growth of Gram-positive *S. aureus* and Gram-negative *E. coli* bacteria. Microstructured surface has been fabricated by the top-down parylene-C etching technique. The adsorption of polyvinylpyrrolidone (PVP)-stabilized SeNPs was tested on naturally hydrophobic, plasma-treated, and positively charged silanized parylene-C surface. Besides, due to the importance of cellular compatibility with the potentially designed antibacterial surface, we used mesenchymal stem cells to let them interact with both flat and microstructured parylene-C surface to evaluate the cellular viability and morphology. Results showed here contribute to the knowledge of the design and development of antibacterial biomedical coatings based on parylene-C.

## Materials and Methods

### Preparation of Flat Parylene-C Film

Parylene-C layer with a thickness of ≈5 µm was deposited on Si wafer according to the following procedure. 5.5 g of parylene-C was deposited on ≈100 mm Si (100) wafer by chemical vapor deposition (CVD). In the next step, wafers with parylene-C layer were cut into (2 × 2) cm^2^ using a semiautomatic dicing saw ESEC 8003.

### Fabrication of Parylene-C Micropillars

**FIGURE 1:**
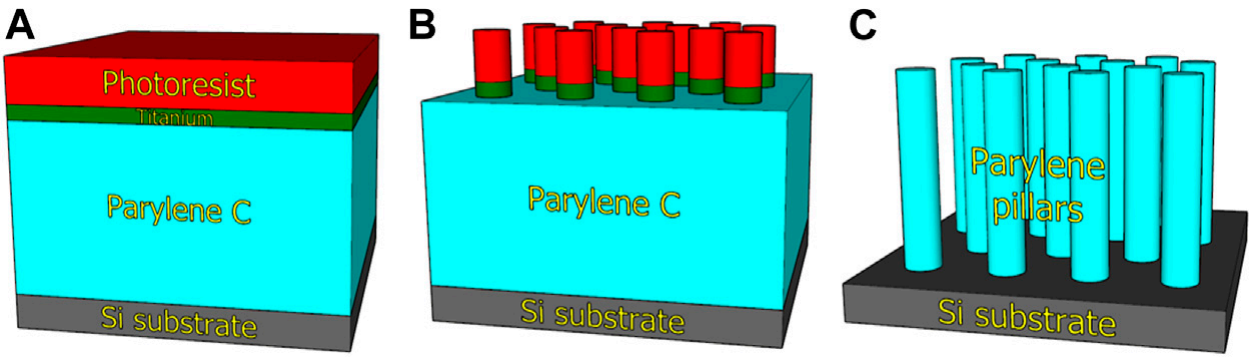
Scheme of micropillar array fabrication by plasma etching of parylene-C. Deposition of parylene-C, titanium, and photoresist on Si wafer **(A)**. Transfer of mask pattern by photolithography and titanium etching **(B)**. Etching of parylene-C to develop micropillar array **(C)**.

The array of micropillars was fabricated *via* a “top-down” process in which 10 µm thick layer of parylene-C was deposited on the Si (100) wafer using the CVD method (Figure 1A). The deposition of an ≈300-nm thick Ti layer using the electron beam evaporation technique was followed by standard UV photolithography *via* a mask aligner in the vacuum contact mode using the photoresist (PR) AZ 5214E (Figure 1B). The thickness of PR was 1.4 µm. PR development created the pattern of hexagonally arranged features with a diameter of 2 µm and a center-to-center distance of 4 µm. Then, titanium was etched using reactive-ion etching (RIE) in Cl_2_ plasma by means of chlorine-based RIE. Finally, the wafer was placed into the ion beam etching (IBE) instrument employing a Kaufman ion-beam source with collimated grids using pure O_2_ plasma for etching the parylene-C from the areas uncovered by Ti. The PR was completely removed during the IBE process, and the Ti residue was additionally removed using chlorine-based RIE (Figure 1C). The last step was wafer cutting into single chips with dimensions of (2 × 2) cm^2^ using femtosecond laser, which eliminates the generation of impurities in comparison to other cutting techniques.

### Synthesis of Selenium Nanoparticles

Colloidal selenium nanoparticles were prepared by wet-chemical reaction (Li et al., 2010). The aqueous solution of 25 mM sodium selenite (Na_2_SeO_3_) was mixed with aqueous solutions of 28 mM L-cystein (C_3_H_7_NO_2_S) and 25 μM PVP under vigorous stirring. When the ruby red color of the mixture developed, it indicated the end of the reaction, and stirring was stopped. Afterward, the colloidal solution was purified three times with deionized water by ultracentrifugation. SeNP solution with final concentration of 0.2 mg·ml^−1^ was subsequently diluted as follows: no dilution (0×), 5×, 10×, 20×, and 50×. All chemicals were purchased from Sigma-Aldrich with a chemical purity grade of per analysis (p.a.). Sodium selenite was used as a precursor of Se ions, L-cystein as a reducing agent, and polyvinylpyrrolidone with an average molecular weight of ≈40,000 g∙mol^−1^ as a stabilizing agent. Nanoparticle morphology and size were characterized by the scanning transmission electron microscope (STEM). Nanoparticle zeta potential (Zetasizer Nano ZS instrument; Malvern Instrument Ltd., United Kingdom) was measured at the condition of 25°C and equilibrating time 0 s. Calculations considering the diminishing of particle concentration were based on the Smoluchowski model, with parameters F (κa) of 1.50. For measurement, disposable cuvettes of type DTS1070 were used. The measurements were performed under the automatic setting of attenuation and voltage selection. All measurements were done in triplicates.

### Decoration of Parylene-C With Nanoparticles

Si squares with parylene film were treated with O_2_ plasma for 1 min to induce parylene film hydrophilicity. Then, a self-assembled monolayer (SAM) of 3-aminopropyltrimethoxysilane (APTES) was deposited using CVD. A 30 µl of APTES was put on the glass slide next to the wafer and heated to 120°C for 30 min in an SAM chamber (custom-made). The adsorption of SeNPs with various dilutions (0×, 5×, 10×, 20×, and 50×) to the parylene film was tested and optimized for bare, plasma-treated, and silanized surface. Si wafer with parylene-C film was immersed into the petri dish with various dilutions of SeNP solution for 90 min. Afterward, the Si wafers decorated with SeNPs were thoroughly rinsed with DI water and air-dried. We named our samples Fc and Mc for control experiments on the flat (F) and micropillar (M) substrate, respectively. SeNP decorated samples were named as F0-F50 and M0-M50 for various dilutions (0×, 5×, 10×, 20×, and 50×).

### Surface Characterization

The parylene-C modification was characterized by measuring the contact angle (CA) by applying a water drop on the surface. The CA was evaluated using Surfaceware 8 software, and the statistical analysis was performed on 5 drops from each step of the modified surface. The distribution of SeNPs on parylene-C was characterized with the scanning electron microscope (SEM), and X-ray photoelectron spectroscopy has been used to analyze surface chemistry (XPS). XPS spectra were analyzed by a peak fitting software (CasaXPS version 2.3.18PR1.0).

### Antibacterial Test

Antibacterial properties of SeNP decorated parylene-C films (flat and micropillars) were further evaluated *via* the colony counting method using Gram-negative bacteria such as *E. coli* and Gram-positive bacteria such as *S. aureus*. Bacterial strains were cultured on Columbia blood agar 5% (Labmediaservis) at 37°C overnight. Bacterial inoculum was prepared by diluting the bacterial colonies in Mueller Hinton broth (Sigma-Aldrich) to cell density 1–2·10^6^ CFU·mL^−1^. The samples were rinsed in sterile water, the bacterial inoculum was spread onto the surface, and the volume of the inoculum was 25 μm·cm^2^. Samples with inoculum were covered by a sterile plastic foil and incubated for 24 h at 37°C. Subsequently, the samples were rinsed in PBS, and adhered bacteria were de-attached by vortexing and sonication. Collected bacterial suspensions were diluted in PBS by decimal dilution, and the colony counting method was used to determine the bacterial count. The suspensions were inoculated on PCA agar plates and incubated for 24 h at 37°C. Bacterial colonies growing on the plates were then counted, and the colony forming unit was calculated (CFU·cm^−2^). The experiment was performed in triplicates. The samples without nanoparticles but plasma-treated were taken as the control. The dimension of each sample was 2 × 2 cm^2^.

### Viability of Mesenchymal Stem Cells

Mesenchymal stem cells (MSCs) isolated during the plastic surgery were cultured in the MSC growth medium (Sigma-Aldrich) supplemented with 5% FBS, 1% penicillin/streptomycin, and 1% L-glutamine. The cells were harvested by trypsinization in 0.25% solution of trypsin/EDTA at 80% confluence. The viability of cells was studied by the tetrazolium-based assay. The proliferation of MSCs on the samples was measured with 2,3-bis-(2-methoxy-4-nitro-5-sulfophenyl)-2H-tetrazolium-5-carboxanilide (XTT) and evaluated on days 1 and 3 after seeding; the initial cell density was 1·10^4^ cells per area. Briefly, the cells were cultured for a definite period and then gently washed twice with preheated PBS. The mixture of 150 ml culture medium and 50 ml tetrazolium dye (XTT, 1 mg·mL^−1^ in PBS, pH 7.4) was added to the samples. After 4-h incubation, the absorbance of the solution was measured at 450 nm. Further, optical microscopy was used to observe the cellular morphology on the designed surfaces. MSCs on the micropillars were fixed with 4% paraformaldehyde in PBS for 15 min, washed twice in buffer solution, and permeabilized in 4% Triton-X100 solution for 1 h. Then, the cells were washed in buffer, and immunostaining of cells was performed. According to the manufacturer, the ready probe ActinGreen (Thermo Fisher) has been applied to stain actin filaments, and the cells were imaged at 480 nm. MSCs on a flat surface have been imaged by the DIC mode of the optical microscope. The cell surface area was measured using ImageJ software. The experiment was performed in triplicates. The samples without nanoparticles but plasma-treated were taken as the control. The dimension of each sample was (2 × 2) cm^2^.

### Statistical Analysis

The data are presented as mean ± standard error. The statistical one-way analysis of variance (ANOVA) with a confidence level of 95% was used.

## Results

### Characterization of Synthetized Selenium Nanoparticles

**FIGURE 2:**
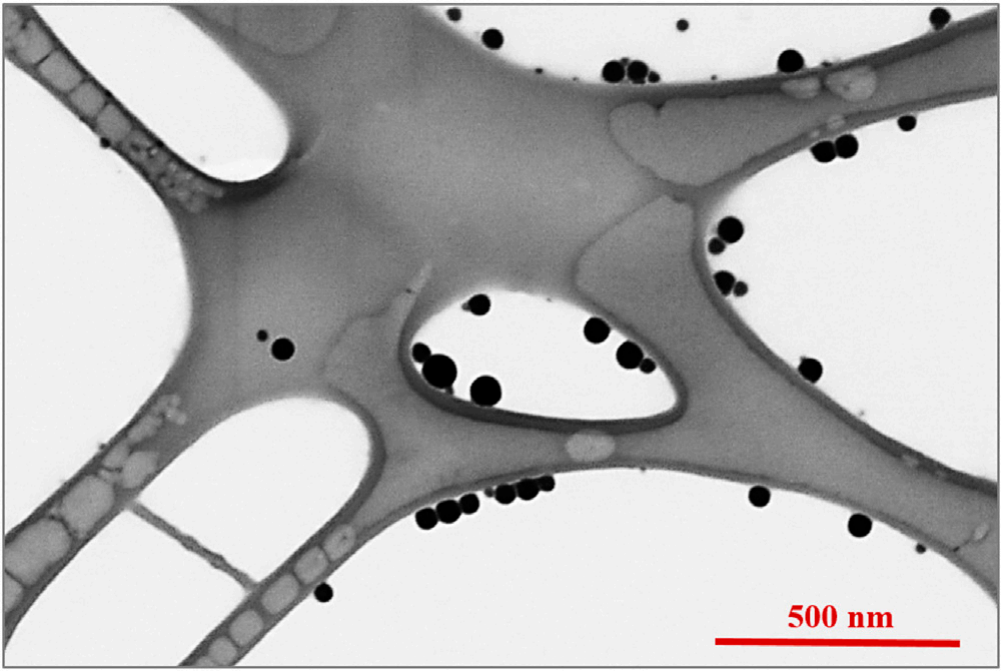
STEM image of selenium nanoparticles.

The morphology and size of SeNPs were determined by a high-resolution STEM with an accelerating voltage of 30 kV. A 10 μl of SeNP colloidal solution was dropped onto a carbon membrane for STEM analysis and dried at an ambient temperature of 22°C. STEM image in Figure 2 presents SeNPs that indicate a uniform spherical shape of nanoparticles with no agglomerations. The average particle size of SeNPs has been estimated (42 ± 7) nm. The stability of SeNPs was analyzed by measuring their electrokinetic zeta (ζ) potential. The measured ζ potential of PVP-stabilized SeNPs synthesized in this work was (−31.6 ± 1.9) mV at pH 6.3 and 25°C.

### Preparation and Characterization of Selenium Nanoparticles/Parylene-C Films

**FIGURE 3:**
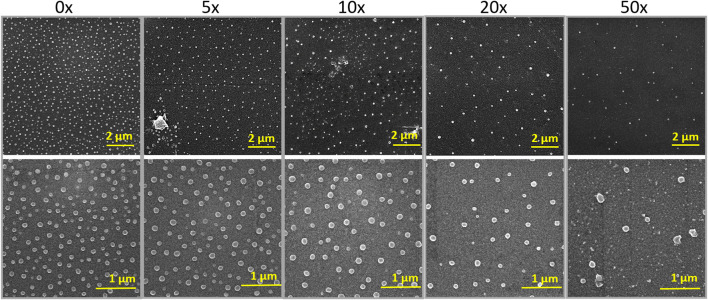
SEM images of SeNP decorated flat parylene-C with the different nanoparticle dilutions shown for two different scale bars.

**FIGURE 4:**
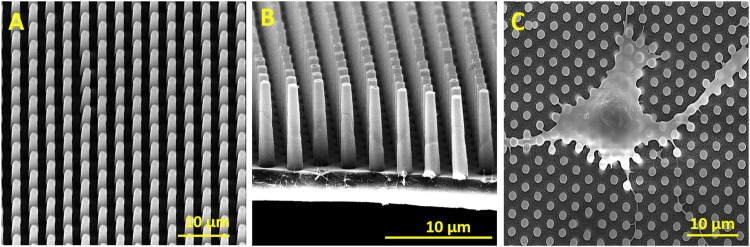
SEM images of the hexagonally ordered micropillars fabricated *via* etching of parylene-C **(A,B)**. The representative SEM image of fixed mesenchymal stem cells laying on the micropillar substrate **(C)**.

**FIGURE 5:**
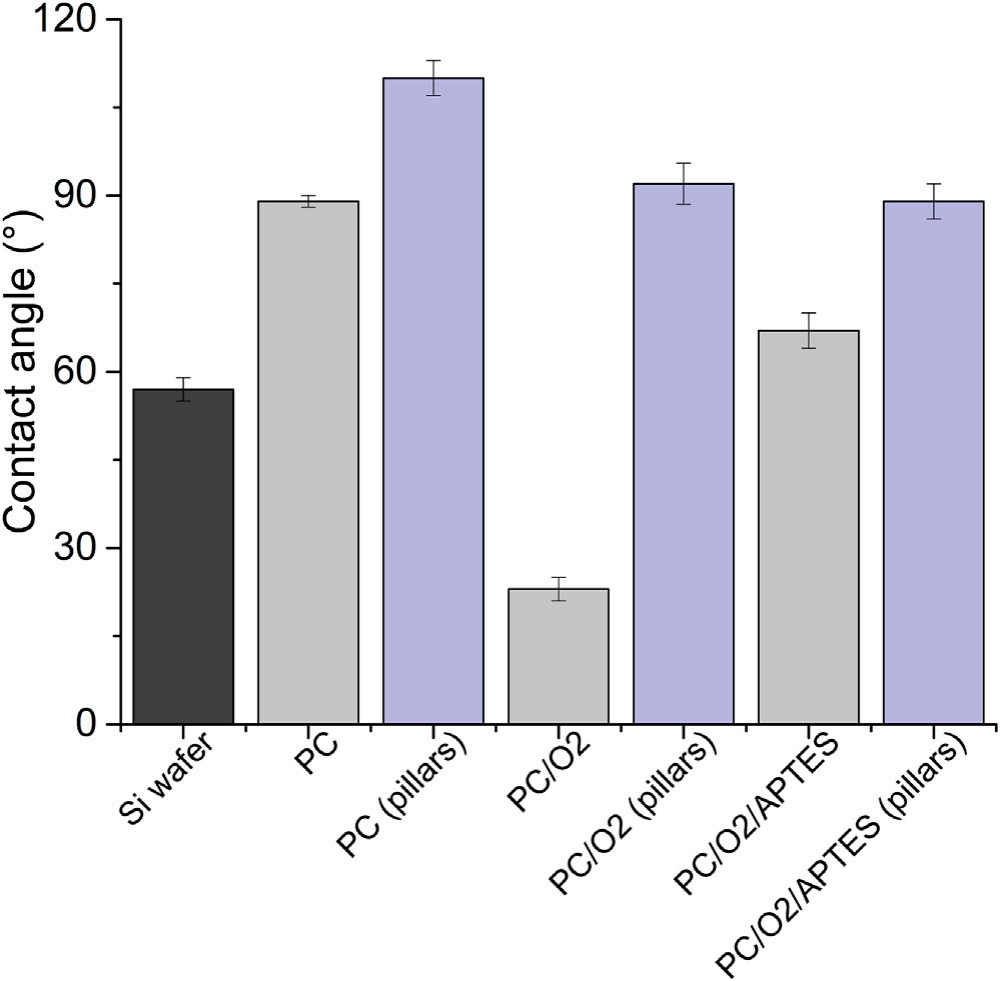
Contact angle measurement of the parylene-C–coated glass slide and micropillar array under the different surface treatment (PC = parylene-C, O_2_ = plasma treatment, and APTES = silane).

**FIGURE 6:**
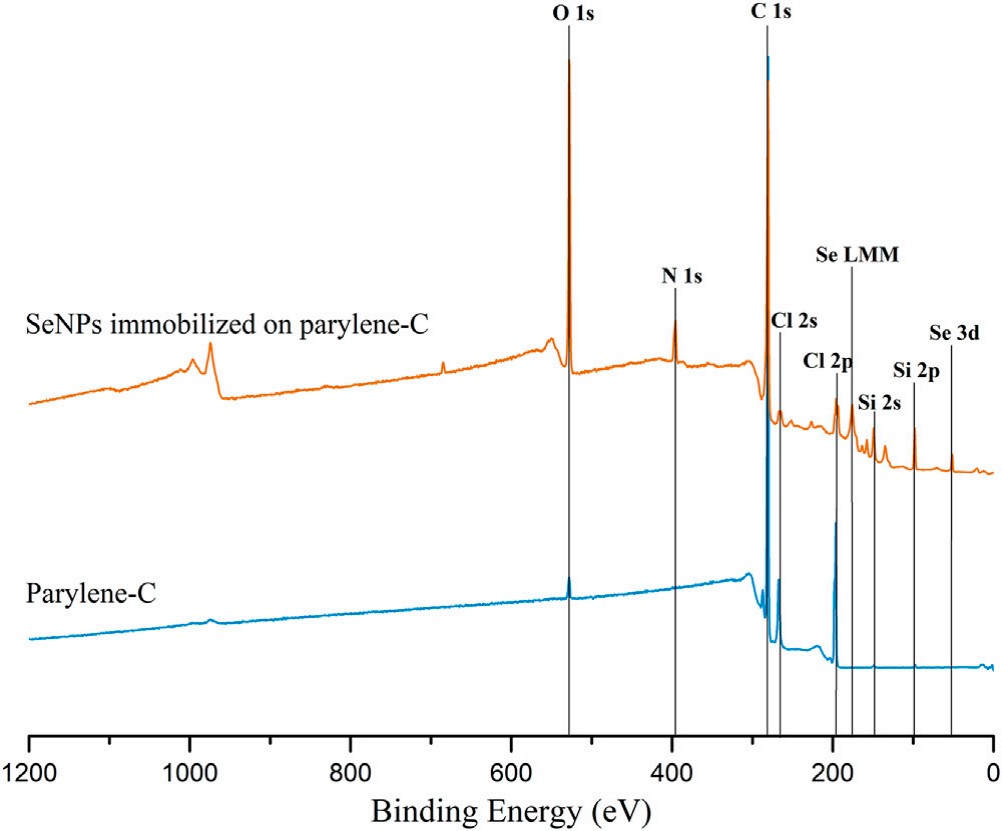
Representative XPS wide spectrum of the flat parylene-C–coated glass slide (blue) and parylene-C surface with immobilized SeNPs *via* APTES silanization.

The decoration of parylene-C films was initially tested by the adsorption of PVP–SeNPs on untreated and plasma-treated flat parylene-C surfaces. The SEM imaging did not show any nanoparticle deposition on such surfaces (data not shown). The surface functionalization of flat parylene-C with amino-terminated silane APTES provided reproducible adsorption of SeNPs as follows: ≈ 12 SeNPs·µm^−2^ (no dilution), ≈9 SeNPs·µm^−2^ (5× dilution), ≈5 SeNPs·µm^−2^ (10× dilution), ≈3 SeNPs·µm^−2^ (20× dilution), and ≈1 SeNPs·µm^−2^ (50× dilution) (Figure 3). The same modification procedure has been performed for microstructured parylene-C. Parylene-C micropillars fabricated by the etching technique of parylene-C provided a micropillar array of the hexagonally ordered parylene-C pillars with ≈2 µm diameter, ≈4 µm center-to-center distance, ≈8 µm length, and cylindrical morphology, as confirmed by a scanning electron microscopy (Figures 4A,B, the representative MSCs are shown on Figure 4C). The parylene-C functionalization was characterized by contact angle measurement (Figure 5). After oxygen plasma treatment, the flat parylene-C film with a contact angle (*CA*) ≈90° dropped to ≈25°. The *CA* value for the highly hydrophobic microstructured parylene-C film dropped down from *CA* ≈110° to 90°. Silanization of the surfaces with APTES increased the *CA* value to ≈65° for the flat parylene-C while remaining unchanged (≈90°) for the microstructured surface. The process of APTES and PVP–SeNP deposition on parylene-C film was confirmed by XPS analysis (Figure 6). The wide spectrum of the flat parylene-C film shows the main peak characteristics of untreated parylene-C (C1s, Cl 2s, and Cl 2p). The spectrum for the APTES/SeNPs/parylene-C sample showed N1s peak from the APTES and PVP stabilizing agent, Si 2s and Si 2p peaks arising from the silane deposition, and Se 3d; Se LMM peaks are characteristic for the selenium nanoparticles. Table 1 summarizes the atomic percentage values of the selected.

**TABLE 1:** Atomic percentage values of the main elements presenting the flat and microstructured parylene-C before and after the modifications as extracted from the XPS spectrum.

Flat parylene-C	O 1s (%)	Se 3d (%)	N 1s (%)	Si 2p (%)
Untreated	0.7	0	0.2	0
APTES and SeNP modification	8.4	0.7	1.8	1.9
**Microstructured parylene-C**	**O 1s (%)**	**Se 3d (%)**	**N 1s (%)**	**Si 2p (%)**
Untreated	0.4	0	0.2	0
APTES and SeNP modification	2.9	0.3	0.8	0.8

### Antibacterial Properties of Parylene-C Films

**FIGURE 7:**
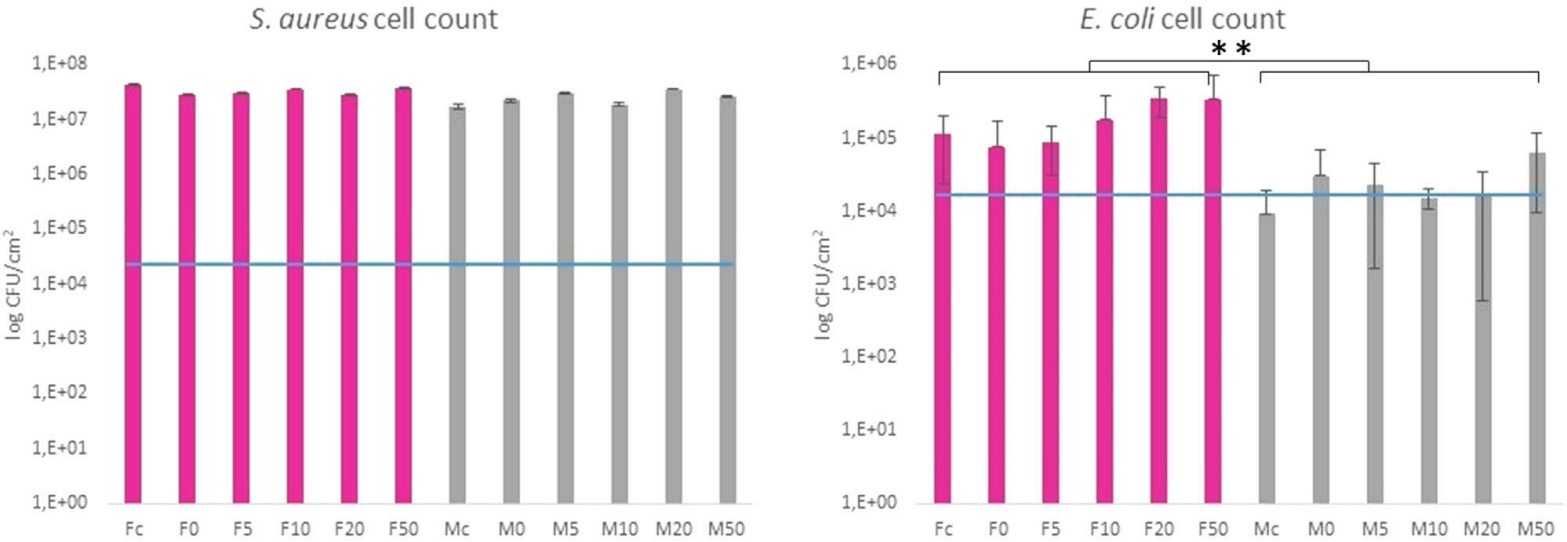
Colony counting method of Gram-positive *S. aureus* and Gram-negative *E. coli* after 24 h incubation with the tested samples. The blue line indicates the initial amount of bacteria deposited on the surface area. Fc and Mc means flat and micropillar surface without nanoparticles (control), respectively. F0-F50 and M0-M50 means nanoparticle dilutions on the flat and micropillar surface, respectively. The ** indicates statistical significance of between groups at *p* ≤ 0.05.

The antibacterial properties of SeNP decorated parylene-C films against Gram-positive and Gram-negative bacteria have been evaluated quantitatively. Figure 7 shows results from the colony counting method for both bacteria after 24 h’ incubation with the designed parylene-C surfaces. The values are related to the initial bacterial density of 2.5·10^4^ CFU·cm^−2^ (blue line) for easier evaluation of antibacterial efficiency. Data showed that the growth of *S. aureu*s was not inhibited on any surfaces, the flat (F; gray color) and microstructured (M; pink color). Unexpectedly, selenium nanoparticles did not enhance the antibacterial efficiency of designed surfaces against *S. aureu*s as was expected. More positive results have been obtained for Gram-negative *E. coli*. By comparing both groups (F and M), we can observe the inhibition effect of bacteria growth on flat parylene-C and the more evident bacteriostatic effect on microstructured parylene-C. However, no significant difference has been observed for SeNP-decorated surfaces (F0-F50 and M0-M50).

### Viability of Mesenchymal Stem Cells on Parylene-C Films

**FIGURE 8:**
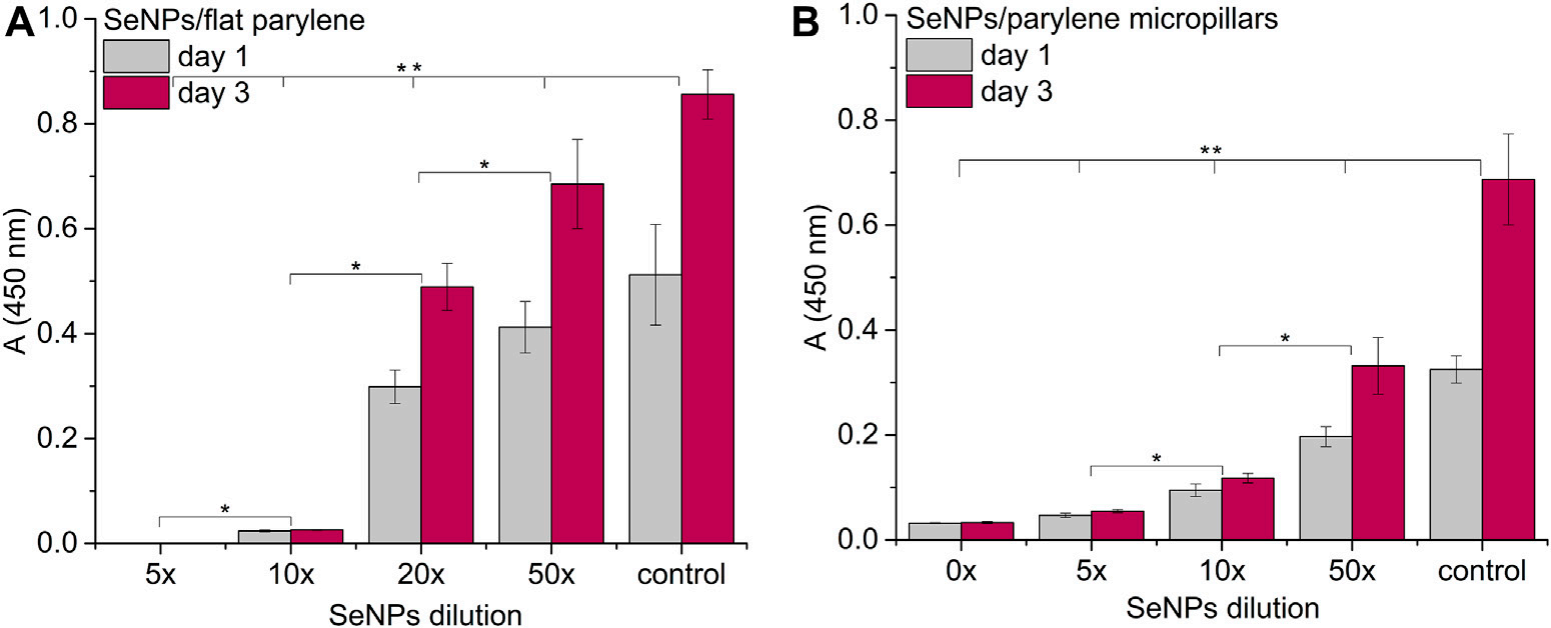
Proliferation assay of MSCs on the SeNP decorated parylene-C flat substrate **(A)** and SeNP decorated parylene-C micropillars **(B)**. The * indicates statistical significance between each dilution at *p* ≤ 0.05 and ** indicates significance between the control and each dilution at *p* ≤ 0.05.

**FIGURE 9:**
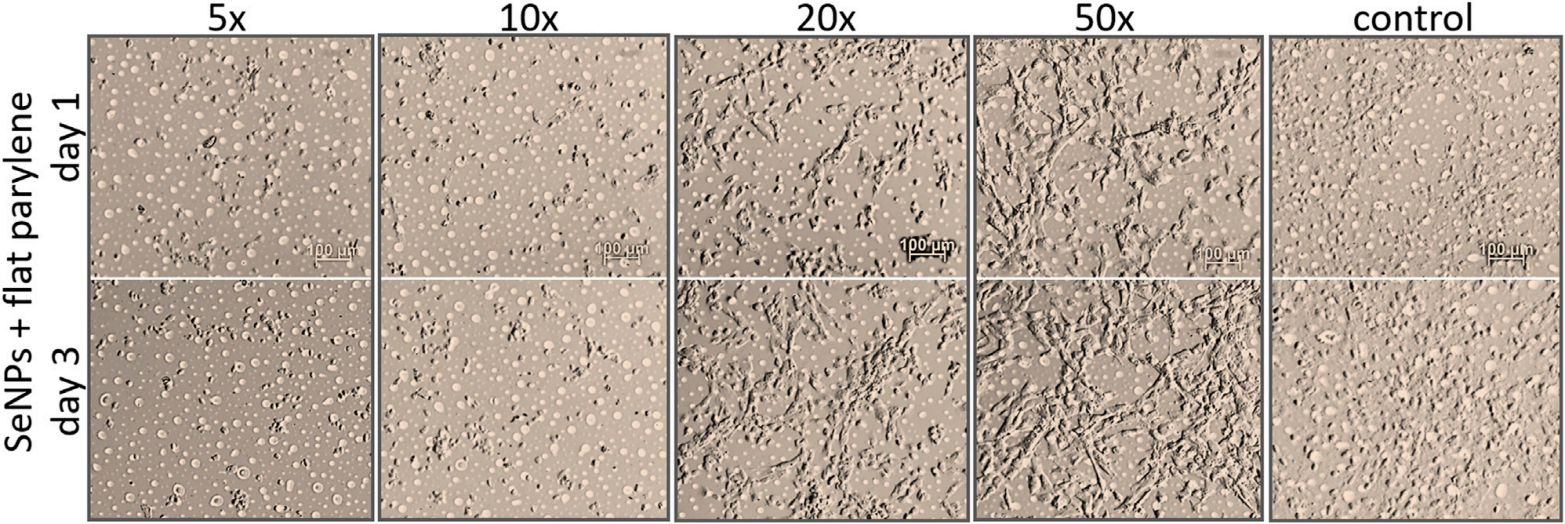
DIC images of adhesion and viability of MSCs on SeNP decorated flat parylene-C film.

**FIGURE 10:**
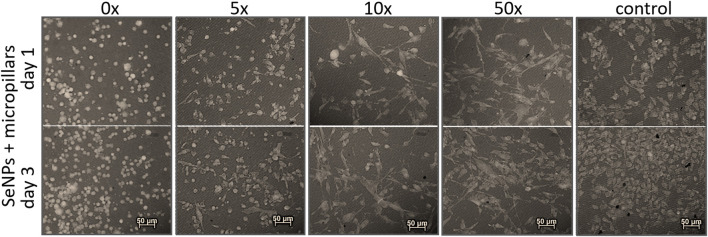
Recolored fluorescence images of adhesion and viability of MSCs on SeNP decorated micropillars.

**FIGURE 11:**
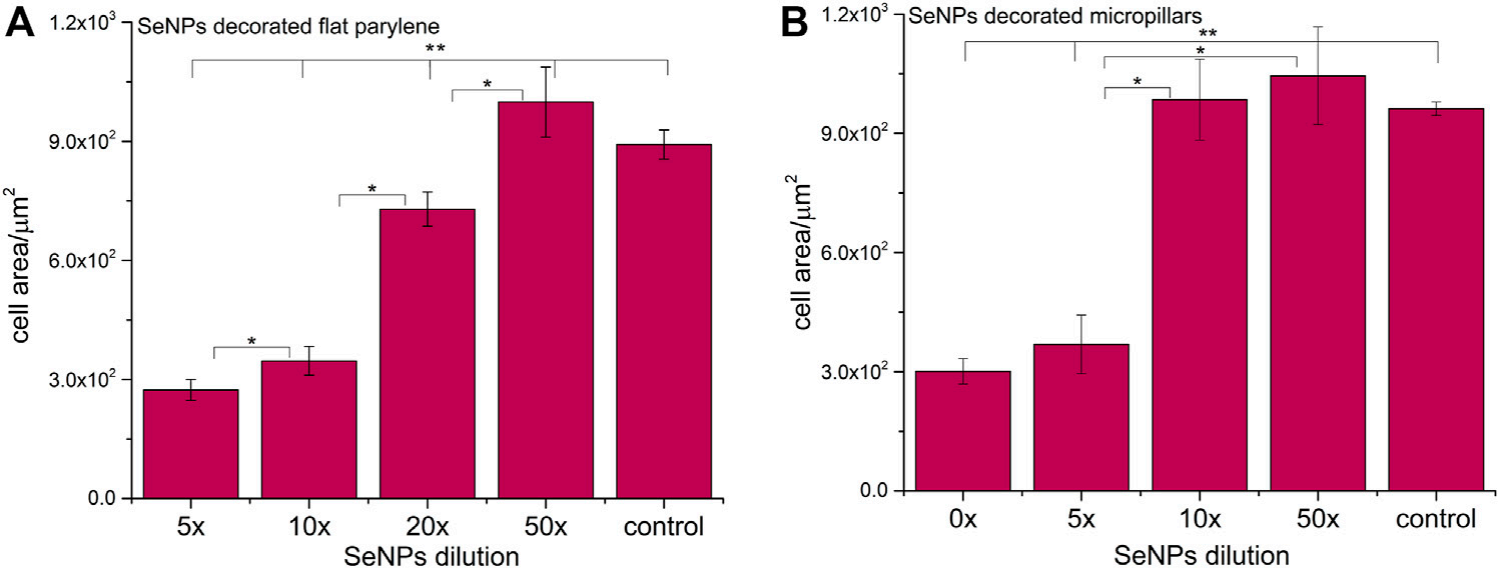
Cell surface area of MSCs cultured on selenium decorated the parylene-C flat substrate **(A)** and SeNP decorated parylene-C micropillars **(B)**. The surface area was measured at day 1. The * indicates statistical significance between each dilution at *p* ≤ 0.05, and ** indicates significance between the control and each dilution at *p* ≤ 0.05.

We performed the XTT test with mesenchymal stem cells to study the viability and proliferation of MSCs on all designed surfaces at day 1 and day 3 (Figure 8). Figure 8A showed a significant decrease in cell adhesion and the proliferation rate on SeNP-decorated flat parylene-C films compared to the undecorated parylene-C “control.” The samples with the lowest nanoparticle dilutions (5× and 10×) exhibited almost 100% killing efficiency to the mesenchymal stem cells even after 24 h. The cytotoxicity/viability of cells was further confirmed by DIC imaging of MSCs by optical microscopy (Figure 9). By looking at the data obtained for the micropillar surface (Figure 8B) and comparing them with the microscopic observation (Figure 10), one can notice that the MSCs adhered and proliferated slowly on microstructures compared to the flat surface. Moreover, 5× dilution was still favorable for the cell survival, and well-spread cells were clearly observed on the captured images. We also measured the cell surface area on each sample (Figure 11). The cell surface area corresponded well with the viability stage of cells.

## Discussion

In this work, microstructured and flat parylene-C film were decorated with SeNPs to evaluate antibacterial effectiveness of such designed surfaces and their biocompatibility to mesenchymal stem cells. SeNPs have been chosen as the widely presented antibacterial agent (Geoffrion et al., 2020; Filipović et al., 2021). The synthesized nanoparticles were characterized by ζ potential and STEM microscopy. ζ potential is used as a parameter for the long-term stability prediction of the colloidal solutions (Lowry et al., 2016). In general, the solution with the absolute value of ζ potential above 30 mV can be considered stable. The ζ potential is also dependent on several factors such as concentration, charge, temperature, and pH. The measured ζ potential of PVP-stabilized SeNPs synthesized in our work has been estimated at about (−31.6 ± 1.9) mV at pH 6.3 and 25°C, which correlates with similar results of PVP-stabilized nanoparticles (Vrandečić et al., 2020). STEM microscopy showed nanoparticles with spherical shape and an average particle size of (42 ± 7) nm (Figure 2).

The nanoparticle deposition on parylene-C films by simple adsorption was first tested for differently treated flat parylene-C films. The strength of adsorption of nanoparticles on the surface depends mainly on the surface chemical properties and the topography of both materials. Initially, we started with the adsorption of PVP–SeNPs on untreated and plasma-treated flat parylene-C surfaces that, to our surprise, did not show any nanoparticle deposition. Therefore, we decided to perform the surface functionalization of parylene-C with amino-terminated silane APTES, and thus provide the positively charged surface to negatively charged PVP–SeNPs at pH 7. This functionalization provided evenly distributed nanoparticle deposition on the flat parylene-C film (Figure 3). The same modification procedure has been performed for the microstructured parylene-C film. However, the SEM images of nanoparticle decorated microstructures did not show any nanoparticles due to the bad contrast even after surface metallization (data not shown).

The functionalization of parylene-C films was characterized by contact angle measurements in each step of the surface modification (Figure 5). The flat parylene-C with a contact angle (*CA*) ≈90° dramatically dropped to ≈25° after oxygen plasma treatment, making the surface hydrophilic. However, the *CA* decrease for the highly hydrophobic microstructured parylene-C was not obvious, dropping down from *CA* ≈110° to ≈90°, making the surface slightly hydrophobic. Silanization of plasma-treated surfaces with APTES exposed the amino group on the parylene-C and the *CA* increased to ≈65°, and remained almost unchanged (≈90°) for the flat and microstructured surface, respectively. The minimal change of *CA* for the microstructured surface could correspond with the fact that just the upper part of pillars has been exposed to oxygen plasma, and thus APTES. Furthermore, the XPS analysis was performed for both films to confirm the process of APTES and PVP–SeNP deposition (Figure 6 and Table 1). Parylene-C is poly (*para*-xylylene), in which chlorine atom substitutes one hydrogen atom. The wide spectrum of the flat parylene-C film shows the main peak characteristics for untreated parylene-C, such as C 1s, Cl 2s, and Cl 2p (Bi et al., 2014). The spectrum for the APTES/SeNPs/parylene-C sample showed that N 1s peak from the APTES and PVP-stabilizing agent, Si 2s and Si 2p peaks coming from the silane deposition and Se 3d, and Se LMM peaks are the characteristics for the selenium nanoparticles. The percentage elemental analysis of the flat and microstructured parylene-C film is summarized in Table 1. The lower values for the microstructured film could correspond with the presence of gaps between pillars. Nevertheless, the XPS analysis confirmed the successful functionalization and decoration of parylene-C surfaces with APTES and SeNPs, respectively.

By finding the successful adsorption of nanoparticles by electrostatic interaction on the silanized surface, we proceeded to prepare representative samples differing in the number of nanoparticles per area. Thus, we made a serial dilution of nanoparticle stock solution from 0× to 50×, and we let the SeNPs interact with the silanized surface for 90 min. The density of nanoparticle coverage was controlled by SEM microscopy (Figure 3). After the optimization of the nanoparticle adsorption, we were able to reproducibly obtain samples with ≈12 SeNPs·µm^−2^ (no dilution), ≈9 SeNPs·µm^−2^ (5× dilution) ≈ 5 SeNPs·µm^−2^ (10× dilution), ≈3 SeNPs·µm^−2^ (20× dilution), and ≈1 SeNPs·µm^−2^ (50× dilution). The SEM images of SeNP decorated parylene-C films also showed that the nanoparticle size differs from that obtained by STEM microscopy. SEM images in Figure 3 estimated the size of nanoparticles about (88 ± 8) nm, which may be due to the sample coating by ≈10 nm layer of gold and a low-resolution SEM imaging. Nevertheless, we managed to reproducibly decorate the nanoparticles on amino-functionalized parylene-C to obtain the representative samples for further antibacterial and cytotoxicity assays.

Parylene-C, as many other medical polymers, does not exhibit any significant antibacterial properties. On the other hand, some inorganic nanoparticles (Ag, Au, Se, *etc*.) possess antibacterial action themselves (Guerrero Correa et al., 2020). Decoration of such polymeric materials with these nanoparticles has been found as one of the strategies for improving the antibacterial properties of polymers. Selenium is the trace element for human health, and thus, SeNPs have become emerging nanomaterials in biomedicine. SeNPs have been shown in many studies as promising antimicrobial agents (Nguyen et al., 2017; Menon et al., 2020). Besides, the decoration of PU, PVC, and silicon (Tran and Webster, 2013), as well as non-polymeric biomaterials (Bilek et al., 2019) with SeNPs, has increased the antibacterial properties of materials toward Gram-negative and Gram-positive bacteria in a relatively positive manner. The suggested correlation between SeNP size and antibacterial action has been studied for the nanoparticle size range between 40 and 205 nm (Huang et al., 2019). The authors showed that the best antibacterial efficiency was found for 81 nm SeNPs. The antimicrobial activity of SeNPs with different surface chemistry and structure has also been studied for several common bacterial strains (Filipović et al., 2021). Moreover, micro- and nanostructuring of hard biomaterials have also been found to have significant impact against the bacteria compared to the non-structuring ones (Li et al., 2019; Liu et al., 2020). Here, we studied the synergistic antibacterial effect of the modified parylene-C polymer with both, selenium nanoparticles and microstructures in the form of micropillars. As for the model of bacterial cells, we chose Gram-positive *S. aureu*s and Gram-negative *E. coli*. Both bacteria differ from the membrane components, and thus, they might have a different response to nano-/micro materials (Epand and Epand, 2009). The presented data in Figure 7 are related to the initial bacterial density of 2.5·10^4^ CFU·cm^−2^ (blue line). It enables one to estimate whether the surface is favorable for bacteria adhesion and growth or it exhibits bacteriostatic or bactericide effect. The growth of *S. aureu*s was not inhibited on any surfaces, the flat (F; gray color) and microstructured (M; pink color), respectively, as it is shown in Figure 7. Unexpectedly, selenium nanoparticles did not enhance the antibacterial efficiency of both surfaces, and more importantly, the significant difference has not been observed even for the series of nanoparticle dilutions. The mechanism of antibacterial action of SeNPs is supposed to damage the cellular membrane and produce reactive oxygen species (ROS). (Huang et al., 2019). The possible explanation for this non-effectivity of SeNPs to act as antibacterial agents can be due to the unsuitable nanoparticle size, membrane composition of Gram-positive bacteria, as well as the positive charge on the un-decorated parylene-C residues, which can attract the bacteria and support them in the growth. Moreover, the fabricated micropillars did not influence *S. aureus* growth, probably due to the “bigger” microstructures and pitches that made the surface favorable to bacteria colonization even in the interpillar space. Considering the *S. aureus* and *E. coli* size of 0.5–1 μm and 1–2 μm, respectively, both bacteria can easily penetrate the free space between pillars, and they may colonize the bottom of the substrate. Another reason for the ineffectiveness of the surface against *S. aureus* may be the stronger cell wall structure of Gram-positive bacteria and the spherical shape of the cocci, which allows them to resist surface irregularities more effectively. However, most of these reasons resulting in the growth of *S. aureus* on these surfaces are probably caused by the combination of two or more factors. A little bit different result was obtained for Gram-negative *E. coli*. Comparing both groups (F and M), one can observe an inhibition effect to bacteria growth on flat parylene-C and a more visible bacteriostatic effect on the microstructured parylene-C. However, within the two groups (F0-F50 and M0-50), no significant difference has been observed. It means that the SeNPs did not act as an antibacterial agent, and more likely, the parylene itself and microstructuring played a more important role in antibacterial action toward Gram-negative bacteria. However, the effect of the positively charged surface from the APTES functionalization of parylene which has been mentioned above has been observed to attract and support the growth of *E. coli* (Sharma and Conrad, 2014). Here, it could contribute to the *E. coli* adhesion but not to the growth, as shown in the graph. More likely, the positive charge had better impact on Gram-positive *S. aureus* adhesion and growth. Our results from the SeNPs’ ineffectiveness to act as an antibacterial agent have also been observed previously (Nguyen et al., 2017). We believe that even if there can be the attraction of negatively charged bacterial membrane to the positively charged surface, the SeNPs, due to the size and repulsive negative charge, cannot penetrate the cell membrane successfully and cause damage. Also, the different membrane compositions of Gram-positive and Gram-negative bacteria can be more or less sensitive to these designed surfaces. It has been published previously that both types of bacteria adhered rapidly on the positively charged surfaces, but with no obvious growth of the Gram-negative bacteria, which can also correlate with our results (Gottenbos et al., 2001). In conclusion, our results highlight the difficulty in understanding the role of bacterial cell surface characteristics to the nanoparticle resistance and biomaterial surface properties, and it must be studied in a more comprehensive way.

The interaction of the specific medical surface with mammalian cells is the key factor in determining material biocompatibility. A variety of biomaterials are designed, for example, to have high efficiency of antibacterial action and do not necessarily exhibit cytocompatibility to living cells. Since the nanoparticles like antibiotics can kill bacteria in a certain lower or higher dose, normal mammalian cells exposed to the same concentration of nanoparticles can also be killed. Therefore, it is important to find some balance between effective antibacterial action and none or low cytotoxicity to normal cells and/or good effectiveness against cancer cells. Moreover, mechanical or physicochemical micro-/nanostructuring of the biomaterial surface has been suggested as one of the key factors determining the cell adhesion, proliferation, differentiation, *etc* (Bilek et al., 2019; Fohlerova et al., 2021). In this work, XTT cytotoxicity and proliferation assay have been performed with mesenchymal stem cells (Figures 8A,B) and confirmed by microscopic imaging of cells on tested surfaces (Figure 9, 10). Quantitative data from the micropillar-based surfaces show more cytotoxic effect toward MSCs than the flat-based samples. However, the image analysis confirmed that MSCs on the microstructured surfaces were just less adhered and slowly proliferated. Furthermore, the morphology of MSCs interacting with the flat parylene-C surfaces of lower SeNP dilutions (5× and 10×) was mostly of rounded shape due to the obvious apoptosis. MSC viability on more SeNP diluted flat samples (20×-50×) looked prolonged in shape, compared to the control sample, not flattened, rectangular, and widespread on the surface, which is shown by the DIC contrast mode (Figure 9). This could be caused by the surface chemical and morphological properties inducing some stress in cells. On the other hand, the 5× dilution of nanoparticles on the microstructured surfaces has still been favorable to the MSC viability. In the 10× diluted sample, the cells were well spread with a prolonged and rectangular shape. MSCs on a control sample are round, widespread cells that can be done by the higher cell density, thus leading to spatial inhibition (Figure 10).

SeNPs have often been studied for their anticancer properties. For example, SeNPs showed a dose-dependent antibacterial effect toward Gram-negative and Gram-positive bacteria and a low cytotoxic effect to dermal fibroblast cells at a range of concentrations up to 1 ppm while showing an anticancer effect toward human melanoma and glioblastoma cells at the same concentration range (Geoffrion et al., 2020). Antibacterial properties of SeNPs and their toxicity to cancerous Caco-2 cells also showed various degrees of toxicity on cells after 24 h of exposure (Nguyen et al., 2017). In our previous work on SeNP decorated titania nanotubes, we showed enhanced antibacterial properties of nanotubes and the toxicity of such surfaces toward non-cancerous and cancerous cells in a dose-dependent response (Bilek et al., 2019). However, our results on SeNP decorated parylene-C samples also showed dose-dependent toxicity to the mesenchymal stem cells. To take it into consideration, intensive optimization and testing of nanoparticle concentration, surface chemistry, size, and morphology must be performed to develop surfaces with desired properties.

## Conclusion

There is a growing demand for the investigation and the development of new potential antibacterial surfaces toward Gram-negative and Gram-positive bacteria and no toxicity to normal mammalian cells. This can be approached by finding the optimal combination of a type of the material, surface chemistry, topography, additional modifications of surface, and dose-dependent utilization of nanomaterials like nanoparticles. Parylene-C is a widely used polymer in biomedicine, but it suffers from low antibacterial action. SeNPs have been proposed as promising antibacterial agents due to their nature for the human body. Here we, for the first time, combine and study the synergistic effect of SeNPs and microstructured parylene-C. We performed the fabrication of parylene micropillars by oxygen plasma etching of parylene-C to introduce and study the antimicrobial effect and biocompatibility of microstructured parylene-C. Our results showed almost no antibacterial effect toward *S. aureus*, while some bacteriostatic effect was observed for *E. coli* on the flat and microstructured parylene. However, SeNPs did not show any improvement in the antibacterial properties of parylene-C samples in dose-dependent experiments. Additionally, mesenchymal stem cells’ interaction with all designed surfaces showed a cytotoxic effect at high selenium nanoparticle concentrations. Our results point out that the microstructuring of parylene-C could be one of the options for improving the antibacterial properties of parylene-C. Further optimization of surface chemistry of parylene-C, changing the SeNP size and shape, or different micro- and nanotopographical features and nanoparticles should be tested in the future.

## Data Availability

The original contributions presented in the study are included in the article/Supplementary Material; further inquiries can be directed to the corresponding author.
